# Seroprevalence of Poliomyelitis Antibodies Among Children Aged 1 to 4 Years Old and Factors Associated With Poliovirus Susceptibility; Mexican Health and Nutrition Survey, 2012

**DOI:** 10.1093/cid/ciy621

**Published:** 2018-10-30

**Authors:** José Alberto Díaz-Quiñónez, José Luis Díaz-Ortega, Pablo Cruz-Hervert, Elizabeth Ferreira-Guerrero, Guadalupe Delgado-Sánchez, Leticia Ferreyra-Reyes, Irma López-Martínez, Belem Torres-Longoria, Rodrigo Aparicio-Antonio, Rogelio Montero-Campos, Norma Mongua-Rodríguez, Lourdes Garcia-Garcia

**Affiliations:** 1 Instituto de Diagnóstico y Referencia Epidemiológicos “Dr Manuel Martínez Báez,”, Mexico; 2 División de Estudios de Posgrado, Facultad de Medicina, Universidad Nacional Autónoma de México (UNAM), Ciudad de México; 3 Centro de Investigación sobre Enfermedades Infecciosas, Instituto Nacional de Salud Pública, Cuernavaca, Mexico; 4 Jefatura de la División de Estudios de Posgrado e Investigación, Facultad de Odontología, Universidad Nacional Autónoma de México (UNAM), Mexico City, Mexico

**Keywords:** poliomyelitis, seroprevalence, survey, Mexico

## Abstract

**Background:**

An essential component of the “Polio Eradication and Endgame Strategic Plan 2013–2018” is the evaluation of population immunity. Mexico introduced the inactivated polio vaccine (IPV) into its routine immunization schedule in 2007 but continued to give trivalent oral polio vaccine OPV twice a year during National Health Weeks through 2016.

**Methods:**

To describe the seroprevalence of poliomyelitis among children one to four years old in Mexico and analyze risk factors for susceptibility. We detected antibodies to poliovirus type 1 by microneutralization test in 966 serum samples randomly selected from the National Health and Nutrition Survey, 2012. We assessed variables associated with susceptibility using multivariable logistic regression.

**Results:**

The overall weighted seroprevalence of the general population was 98.39% (95% confidence interval [CI] 96.76–99.21). We found significant differences of prevalence according to age (94.39%, 95% CI 87.56–97.58; 99.02%, 95% CI 95.68–99.79; 99.82%, 95% CI 98.77–99.98; and 100% among children 1, 2, 3, and 4 years old respectively) and number of IPV doses (96.91%, 95% CI 90.55–99.44; 100%; 97.85%, 95% CI 94.46–99.18; and 99.92%, 95% CI 99.45–99.98 for 1 2, 3, and 4 number of doses, respectively). Multivariate analyses showed that susceptibility was associated with younger age, fewer doses of IPV, and certain socioeconomic levels.

**Conclusions:**

Overall seroprevalence was high. However, we found susceptible children among younger ages and children with fewer or unknown IPV doses belonging to certain socioeconomic strata. Results are relevant for countries transitioning from OPV to IPV and underline the importance of achieving high coverage with IPV.

The globally-sustained use of the 2 polio vaccines available to prevent paralytic poliomyelitis—the inactivated poliomyelitis vaccine (IPV) and the oral poliomyelitis vaccine (OPV)—has significantly decreased the incidence of this illness since the World Health Assembly declared its intent to eliminate poliomyelitis in 1988. The main risk for polio today is concentrated in the 3 countries that are still endemic (Afghanistan, Pakistan, and Nigeria) and in some countries where there may be instability in maintaining national health systems, resulting in low vaccine coverage and weak epidemiologic surveillance [1, 2]. In industrialized countries where it has been possible to control the disease, the risk is less; however, poliomyelitis will not be globally eradicated as long as the virus still circulates in any part of the world [3–5]. In the year 2013, the Global Poliomyelitis Eradication Initiative published the “Strategic Plan for the Eradication of Polio: 2013–2018” to consolidate the eradication and containment of all wild-type polioviruses (WP), as well as attenuated polioviruses contained in OPV (Sabin and OPV-like) and those developed from the continued circulation of attenuated poliovirus from the OPV vaccine, known as vaccine-derived polioviruses [6]. The elimination of vaccine-derived polioviruses, as well as other OPV-like viruses, depends on the cessation of use of OPV. The international community agreed to remove the Sabin 2 virus from trivalent OPVs starting in 2016, facilitated by the introduction of at least 1 dose of IPV and completing the schedule with 1 or 2 doses of bivalent OPV (bOPV). The experience developed from trivalent OPV cessation will contribute to the success of those future efforts directed at the cessation of all OPVs.

In Mexico, IPV is used as part of a pentavalent vaccine that has also included diphtheria and tetanus toxoids, a fraction of acellular pertussis and polysaccharide b of *Haemophilus influenzae* since 2007. As a part of the routine vaccination scheme, children are given 1 dose each at 2, 4, 6, and 18 months of age. Additionally, all children between 6 months and 4 years of age that have received at least 2 doses of IPV receive 1 dose of OPV (currently bOPV, since 2017) during the National Health Weeks that are carried out twice a year.

Determining the specific antibodies that are effective against vaccine-preventable diseases is the most precise way to evaluate vaccination programs and has the aim of identifying areas of opportunity, eliminating the risk of outbreaks, and guaranteeing control, elimination, and eradication of the disease [7, 8]. In this study, we used serum samples from the National Health and Nutrition Survey 2012 (ENSANUT) to estimate the prevalence of antibody titers against poliovirus 1 among children 1 to 4 years of age and to identify the risk factors associated with poliovirus susceptibility.

## MATERIALS AND METHODS

ENSANUT 2012 was a cross-sectional, probabilistic and cluster household survey with representative national, regional, urban–rural, and state levels of the civilian, non-institutionalized population that was conducted by the Mexican Secretariat of Health between October 2011 and May 2012. A sample of 50528 households, of an estimated 29429252 households nationwide, was obtained between October 2011 and May 2012, with a total of 96 031 recruited individuals; a blood sample was obtained on 37% of those recruited, excluding children younger than 1 year old. The research design and details of the sample size and sampling design have been described elsewhere [9]. Our study consisted of a secondary analysis based on 966 individuals aged 1 to 4 years who were randomly selected from this age group of the ENSANUT 2012. We obtained sociodemographic characteristics and vaccination history from questionnaires on household information and use of public services and health services.

### Serum Sample Analysis

Antibodies against polioviruses were measured by microneutralization assay using cell culture HEP-2 (Human Epidermoid cancer cells), formerly recommended for routine use in accordance with the World Health Organization (WHO) method [10]. After serum samples’ inactivation at 56°C for 30 min, 2-fold serial dilutions were performed and then the samples were incubated for 1 hour at 37°C with a virus suspension (100TCID 50) of each virus in a 96-well microculture plate. Subsequently, a cell suspension was added to each well and, for the next 6 days, the appearance of cytopathic effect was examined by a standard microscope. Cellular obliteration and plaque formation indicated a low neutralizing antibody concentration; cells are protected where the antibody titer was ≥1:8 [11]. Then, the plates were fixed and serum absorbency was evaluated on an enzyme-linked immunosorbent assay reader.

### Covariates

We included the following covariates: sex of the child, urban/rural residence, region (Northern, Central, Southern, and Mexico City), household materials, and access to Social Security (yes vs no). We also included the number of IPV doses, as registered in the child′s vaccination card or another confirmatory document or referred by the child’s caregiver. We did not obtain the number of OPV doses, since this vaccine is not registered. We used a standard socioeconomic index developed in Mexico on the basis of various household characteristics, including building materials, number of rooms, basic service infrastructure, and ownership of domestic appliances. This index was selected to allow comparison with previous surveys in Mexico [12].

### Statistical Analysis

We calculated the prevalence (95% confidence interval [CI]) of antibodies by demographic and socioeconomic variables and previous vaccination characteristics. We excluded 40 children with no IPV doses and 23 children whose vaccination status was unknown from bivariate and multivariate analyses. We conducted logistic regression models for susceptibility, adjusting for pertinent covariates. Results were expressed as adjusted odds ratios (ORs) with their corresponding 95% CIs. Prevalence estimates and logistic regression models considered sampling weights, using STATA 12 survey data commands for complex surveys.

### Ethical Considerations

The ENSANUT protocol and the specific protocol to analyze sera were approved by the Committees of Ethics, Biosafety and Research of the Instituto Nacional de Salud Pública. Informed consent was obtained from the parents or guardians of children. The collection and management of data were carried out under confidentiality clauses according to Mexican regulation.

## RESULTS

Serum samples of 980 children from 1 to 4 years of age were tested for antibodies against poliovirus 1. Antibody levels higher than 1:8 were found in 966 individuals, with an overall seropositivity of 98.39% (95% CI 96.76–99.21). The prevalence (95%CI) of antibodies by demographic and socioeconomic variables and previous vaccination characteristics are shown in Table 1. There were differences according to age (94.39%, 95% CI 87.56–97.58; 99.02%, 95% CI 95.68–99.79; 99.82%, 95% CI 98.77–99.98; and 100% among children 1, 2, 3, and 4 years old, respectively) and number of IPV doses (96.91%, 95% CI 90.55–99.44; 100%; 97.85%, 95% CI 94.46–99.18; and 99.92%, 95% CI 99.45–99.98 for 1, 2, 3, and 4 doses, respectively).

**Table 1.** Prevalence to Poliovirus Type 1 Antibodies According to Sociodemographic Characteristics and Vaccination History, National Health and Nutrition Survey 2012

Characteristics of Studied Population	Number of Children With Titers ≥ 8	Weighted Population of Children With Titers ≥ 8	Prevalence (%)	95% Confidence Interval	*P* Value^a^
Age in years					
1	143	1944302	94.39	87.56–97.58	<.001
2	231	2204441	99.02	95.68–99.79	
3	301	2228553	99.82	98.77–99.98	
4	291	2279215	100	...	
Sex					
Female	498	4302379	99.32	98.18–99.75	.05
Male	468	4354133	97.5	94.08–98.96	
Residence					
Urban	548	6421828	98.27	96.02–99.26	.647
Rural	418	2234684	98.73	96.46–99.55	
Inactivated polio vaccine doses (vaccination card or memory)					
1	103	1107088	96.91	90.55–99.03	.007
2	18	185072	100	...	
3	178	1811577	97.85	94.46–99.18	
4	609	5100858	99.92	99.45–99.98	
Access to Social Security					
No	198	2173547	97.21	89.77–99.28	.269
Yes	768	6482966	98.79	97.57–99.40	
Socioeconomic level					
1 (Higher)	37	556586	99.46	96.10- 99.93	.05
2	108	1198601	99.28	94.96–99.90	
3	159	1414970	95.73	85.54–98.84	
4	205	1537873	97.16	92.53–98.95	
5 (Lower)	457	3948482	99.46	97.50–99.89	
Region					
Mexico City	15	331443	100	...	.101
Nothern	190	2079158	96.23	89.48–98.71	
Central	346	3524623	99.73	98.83–99.94	
Southern	415	2721289	98.19	95.46–99.29	

^a^Chi-square test.

### Susceptibility

We found 14 susceptible children with titers below 1:8. Of them, 9 were 1 year of age; 4 did not have a known previous vaccination status; 4 had received only 1 dose of IPV; and 5 children had received 3 or more IPV doses according to their vaccine cards. The overall susceptibility rate was 1.06% (95% CI 0.79–3.23). The design of the survey allowed us to estimate that there were 141129 susceptible children nationwide aged 1 to 4 years old. Of these children, most of them were 1 year old (number = 115495). Susceptibility among children 1 year old was 5.61% (95% CI 2.42–12.44).

### History of Vaccination With Inactivated Polio Vaccine

There were 25 children whose vaccination status was unknown. Of these, 5 did not have protective antibodies (these 5 children are included in the paragraph above). Additionally, there were 40 children for whom no IPV vaccine had been registered or the mother had lost the card and stated that the child had not been vaccinated. All of these children had protective levels of antibodies. All the rest of the children had received 1–4 doses of the IPV vaccine.

### Multivariate Analyses

By multivariate analyses, younger age, fewer IPV doses, and certain socioeconomic strata were found to be associated with poliovirus susceptibility (Table 2).

**Table 2.** Variables Associated With Susceptibility to Poliovirus Type 1 by Multivariable Logistic Regression Model, National Health and Nutrition Survey 2012

Variables	Adjusted Odds Ratio	95% Confidence Interval		*P* Value
Region				
Mexico City	...^a^	...	...	...
Northern	2.38	0.44	12.89	.312
Center	0.18	0.01	3.44	.251
Southern	Reference			
Age (years)				
1	Reference			
2	0.01	0	0.18	.001
3	0.07	0.01	0.89	.041
4	...^a^	...	...	...
Boys	1.77	0.28	10.97	.541
Number of inactivated polio vaccine doses				
1	Reference			
2	...^a^	...	...	...
3	0.6	0.13	2.69	.501
4	0.02	0	0.44	.012
Socieconomic level				
1 (highest)	Reference			
2	33.42	0.6	1855.85	.087
3	14.92	1.19	187.57	.036
4	59.5	3.42	1036.62	.005
5 (lowest)	...^a^	...	...	...
No access to Social Security	...^a^	...	...	...

^a^All subjects had poliovirus antibodies.

## DISCUSSION

The present study documents a high seroprevalence of antibodies at a national level (98.39%, 95% CI 96.76–99.21). Susceptibility was associated with younger age, fewer or an unknown number of IPV doses, and certain socioeconomic statuses.

Our study can be compared to 2 previous Mexican surveys, conducted in 1987 and 2000, in which samples were tested by the same technique, allowing comparability between studies. In the 1987 survey, the prevalence of antibodies against the 3 serotypes (1, 2, and 3) was detected in 5260 serum samples in a population aged 1 to 5 years. The levels of immunity were as follows: type 1, 89.9%; type 2, 97.6%; and type 3, 85.4%. Verbal vaccination reports of 3 or more doses showed significant differences. Vaccinated individuals had seroprevalences of 92.7%, 98.6%, and 88.8% for type 1, 2, and 3, respectively; whereas non-vaccinated individuals had seroprevalences of 80.6%, 94.1%, and 74.1%, respectively [13]. Seroprevalences in these children, who were theoretically unvaccinated, could be attributed to subclinical infection, doses not recorded on the vaccination card, or to the environmental transmission of the vaccine poliovirus, considering that at that time only the OPV vaccine (Sabin) was in use in the country. In the year 2000, the presence of antibodies against poliovirus 1 was studied by nationally collecting samples from 6270 children aged 1 to 9 years [14]. Their seropositivity was 99.3% (95% CI 99.1–99.7). Risk factors associated with poliovirus susceptibility were illiteracy (OR 1.5; *P* = .002) and low household income (OR 1.4; *P* = .0487), while a protective factor was access to Social Security (OR 0.41; *P* = .04). Our results show that the prevalence of antibodies continues to be high. However, we found that incomplete schemes, age, and certain socioeconomic strata were associated with poliovirus susceptibility. Our results more likely reflect problems in infrastructure and the performance of health services, which cause shortages of IPV and make access to health services difficult.

A high level of seropositivity against poliovirus 1 among Mexican children aged 1 to 4 years represents an indicator of success of the vaccination program in the country. We tested for serotype 1, given that in a hypothetical case of the importation of wild strains, this serotype would be the most likely to be found, since it has caused recent outbreaks worldwide. There were only 14 cases without detectable antibodies, which means the susceptibility of the studied population was 1.06% (95% CI 0.79–3.23). Although susceptibility is low, the design of ENSANUT 2012 allowed us to estimate that there may have been 141129 susceptible children nationwide aged 1 to 4 years old. Of concern was the fact that most of them were 1 year old (estimated number of unvaccinated 1-year-old children = 115495) and that susceptibility among this age group was 5.61% (95% CI 2.42–12.44).

There has been a global concern regarding shortages of IPV [15]. WHO has devised several strategies, including WHO’s 28-day open-vial policy [16]—which allows the use of a multidose vial for up to 28 days if the cold chain has been properly maintained—and replacing the single, 0.5mL intramuscular IPV dose with fractional doses of 0.1 mL given intradermally [17]. Research indicates that 2 intradermal doses might be more immunogenic against the type 2 poliovirus than a single intramuscular dose [18, 19] and would allow the vaccination of more than twice the number of infants with the same number of vials.

We found 5 children with negative serum samples who had been vaccinated with IPV 3 or more times, according to their vaccine cards. This data attest to the low frequency of vaccination failure. An explanation for their negative samples could be administrative problems, such as the interruption of cold chains, which is improbable since most of children with 1 or 2 doses had adequate levels of antibodies. These cases might also have been due to infrequent cases of failure of humoral immunity [20]. However, we do not have enough data to support this hypothesis.

We identified 40 children with no previous vaccinations, all of them with protective titers. The transmission of OPV from vaccinated contacts during National Health Weeks might explain the immunity among non-vaccinated contacts. Therefore, if these children were indeed not vaccinated with IPV, they might continue to go unvaccinated when OPV ceases to be used.

We also found that certain socioeconomic levels were not associated with poliovirus susceptibility: the highest and the lowest levels harbored protective levels of antibodies. In Mexico, the more underprivileged population receives conditional cash transfers that require that the child and the rest of the family have complete vaccination schedules, while people with stable jobs or a higher socioeconomic status probably are vaccinated through social or private health security. Of concern are the middle layers: individuals who are not affiliated with any Social Security system and who do not benefit from conditional cash transfers.

This study is limited by its cross-sectional design, which reduced our ability to make causal inferences. Additionally, there may have been biases in the collection of information on previous IPV doses, since not all caregivers showed the vaccine card. However, the trending prevalence of protective antibodies is consistent with our hypothesis that fewer doses were associated with poliovirus susceptibility. The main strength of this study is its large sample size, which allowed us to evaluate possible confounders in the statistical models and to provide evidence useful at the national level.

The Polio Eradication and Endgame Strategic Plan 2013–2018 represents a major milestone and requires action on several fronts. It describes specific steps to be followed systematically to successfully achieve its purpose. Although Mexico has been successful in many of these aspects, including attainment of high vaccination coverage, this study highlights possible problems that might arise in low- and medium-income countries transitioning to the exclusive use of IPVs.

## Notes

***Acknowledgments.*** The authors thank Dr Mercedes Yanes Lane for support in translation of the document.

***Financial support.*** This work was supported by the Mexican Council of Science and Technology (grant number 182085 S0008- FONSEC SSA/IMSS/ISSSTE 182085).

***Supplement sponsorship.*** This article appears as part of the supplement “Polio endgame and beyond: Vaccine choices, transmission dynamics, and surveillance implications,” sponsored by The Bill & Melinda Gates Foundation.

***Potential conflicts of interest***. L. G.-G. received grants from the Bill & Melinda Gates Foundation/Stanford University and from GlaxoSmithKline Biologicals unrelated to this report. All other authors report no potential conflicts. All authors have submitted the ICMJE Form for Disclosure of Potential Conflicts of Interest. Conflicts that the editors consider relevant to the content of the manuscript have been disclosed.
